# Salmon Nasal Cartilage-Derived Proteoglycans Alleviate Monosodium Iodoacetate-Induced Osteoarthritis in Rats

**DOI:** 10.3390/md22110507

**Published:** 2024-11-08

**Authors:** Inae Jeong, Jinbum Park, Shinjung Park, Tatuya Wada, Dae Soo Lim, Ok-Kyung Kim

**Affiliations:** 1 Division of Food and Nutrition, Chonnam National University, Gwangju 61186, Republic of Korea; wjddlsdo2@jnu.ac.kr (I.J.); pabre96@jnu.ac.kr (J.P.); qkrtlswjd1@jnu.ac.kr (S.P.); 2 Nihon Pharmaceutical Co., Ltd., Tokyo 103-0012, Japan; tatuya.wada@nihon-yakuhin.co.jp; 3 Daedeok Pharma Co., Ltd., Suwon-si 16226, Republic of Korea; ddpmhwk@hanmail.net; 4 Human Ecology Research Institute, Chonnam National University, Gwangju 61186, Republic of Korea

**Keywords:** osteoarthritis, proteoglycans, joint cartilage, inflammation

## Abstract

Osteoarthritis is a chronic inflammatory condition characterized by the degeneration of joint cartilage and underlying bone, resulting in pain, swelling, and reduced mobility. This study evaluates the efficacy of salmon nasal cartilage-derived proteoglycans in mitigating osteoarthritis symptoms and investigates the underlying molecular mechanisms. This study employed a rat model of osteoarthritis induced by monosodium iodoacetate (MIA) injection. The rats were orally administered salmon nasal cartilage-derived proteoglycans or ibuprofen. Key aspects of osteoarthritis pathology, including impaired exercise ability, inflammation, extracellular matrix degradation, and chondrocyte apoptosis, were assessed using histological analysis, micro-CT, treadmill testing, serum assays, and mRNA/protein expression studies. The MIA injection caused significant cartilage damage, reduced bone mineral density, and impaired exercise ability. Additionally, it elevated serum levels of prostaglandin E2 and nitric oxide, increased the mRNA and protein levels of inflammation-related factors, and activated apoptosis signaling pathways in cartilage. Treatment with salmon nasal cartilage-derived proteoglycans significantly improved cartilage morphology and mineralization, reduced inflammation, and inhibited apoptosis signaling pathways, with effects comparable to those observed with ibuprofen treatment. These findings highlight the potential of salmon nasal cartilage-derived proteoglycans as a therapeutic agent for managing osteoarthritis by effectively reducing inflammation, preventing cartilage degradation, and inhibiting chondrocyte apoptosis.

## 1. Introduction

Arthritis is a chronic inflammatory condition characterized by the degeneration of joint cartilage and underlying bone, resulting in pain, swelling, and reduced mobility. This prevalent disorder affects millions of individuals worldwide, particularly the elderly population [1,2]. There are several types of arthritis, with osteoarthritis and rheumatoid arthritis being the most common. The etiology of arthritis involves a complex interplay of genetic, environmental, and lifestyle factors, including age, obesity, joint injury, and repetitive stress on the joints [3,4,5].

Articular cartilage is a specialized connective tissue that covers the ends of bones in synovial joints, providing a smooth, lubricated surface for articulation and facilitating load transmission with minimal friction [6]. The integrity of articular cartilage is maintained through a dynamic balance between the synthesis and degradation of its extracellular matrix components. Chondrocytes, the primary cells in cartilage, produce and maintain the extracellular matrix, which predominantly consists of collagen and proteoglycans, along with other non-collagenous proteins and glycoproteins [7,8]. In osteoarthritis, this balance is disrupted, leading to progressive articular cartilage degradation and joint dysfunction. Articular cartilage degradation involves multiple factors, including inflammatory processes, matrix metalloproteinase (MMP) activities, and chondrocyte apoptosis. Inflammatory cytokines play a crucial role in both the initiation and progression of osteoarthritis by stimulating the production of MMPs, which degrade extracellular matrix components, particularly collagen, leading to the breakdown of articular cartilage [9,10]. Furthermore, the sustained inflammatory environment induces oxidative stress and promotes chondrocyte apoptosis. The progressive loss of cartilage results in joint stiffness and pain, hallmark symptoms of osteoarthritis [11].

Proteoglycans, essential components of the extracellular matrix in cartilage, are composed of a core protein with covalently attached glycosaminoglycan chains. They contribute to the structural and functional integrity of cartilage by providing compressive strength [12]. Recent studies have highlighted the potential therapeutic effects of proteoglycans in managing arthritis due to their anti-inflammatory and chondroprotective properties. Particularly, salmon nasal cartilage-derived proteoglycans have garnered significant attention for their potential to alleviate osteoarthritis symptoms [13,14]. In a study by Tomonaga et al. [13], oral administration of salmon nasal cartilage-derived proteoglycan at a dose of 10 mg/day for 16 weeks demonstrated chondroprotective effects in subjects experiencing knee joint discomfort. However, the precise mechanisms through which salmon nasal cartilage-derived proteoglycans exert their beneficial effects on osteoarthritis remain to be fully elucidated. This study aims to evaluate the efficacy of salmon nasal cartilage-derived proteoglycans in mitigating osteoarthritis symptoms and to explore the underlying molecular mechanisms. Using an experimental animal model of osteoarthritis, key aspects of osteoarthritis pathology, including impaired exercise ability, inflammation, extracellular matrix degradation, and chondrocyte apoptosis, were evaluated. By elucidating these effects, this study provides a comprehensive understanding of the potential therapeutic benefits of salmon nasal cartilage-derived proteoglycans in managing osteoarthritis.

## 2. Results

### 2.1. Oral Administration of Salmon Nasal Cartilage-Derived Proteoglycans Ameliorates Histological and Mineralization Impairments and Improves Exercise Ability in MIA-Injected Rats

**Figure 1:**
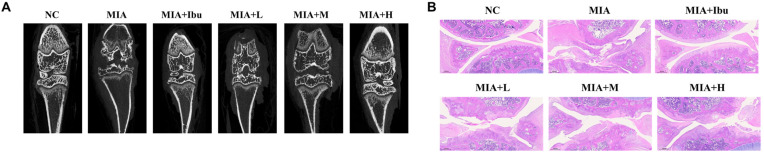

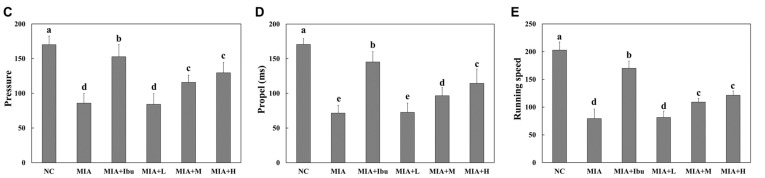
Effects of salmon nasal cartilage-derived proteoglycans on morphological and histological alterations ((**A**) micro-CT; (**B**) H&E staining) and treadmill metrics, specifically the pressure (**C**), propel (**D**), and running speed (**E**), in rats with MIA-induced osteoarthritis. Normal control (NC): normal diet; MIA: normal diet + MIA injection; MIA+Ibu: diet containing 20 mg/kg/bw ibuprofen + MIA injection; MIA+L: diet containing 2.1 mg/kg/bw salmon nasal cartilage-derived proteoglycans + MIA injection; MIA+M: diet containing 4.2 mg/kg/bw salmon nasal cartilage-derived proteoglycans + MIA injection; MIA+H: diet containing 8.4 mg/kg/bw salmon nasal cartilage-derived proteoglycans + MIA injection. Data represent the mean + standard deviation (SD). Different letters (a > b > c > d > e) indicate statistically significant differences between groups (*p* < 0.05), as determined using Duncan’s multiple range test.

Morphological analysis of the knee joints of rats with monosodium iodoacetate (MIA)-induced osteoarthritis revealed significant changes, including fibrillation, fissures, and cartilage degradation (Figure 1A,B). MIA injection also resulted in reduced bone mineral density, bone volume/total tissue volume, trabecular number, and trabecular thickness, along with increased trabecular separation, as evidenced by micro-CT analysis (Table 1). However, MIA-injected rats treated with ibuprofen or salmon nasal cartilage-derived proteoglycans exhibited fewer morphological changes and improved mineralization metrics. Specifically, these treatments enhanced bone mineral density, bone volume/total tissue volume, trabecular number, and trabecular thickness, while decreasing trabecular separation (*p* < 0.05; Table 1, Figure 1A,B).

Furthermore, the MIA-injected rats exhibited significantly decreased pressure, propel, and running speed on the treadmill compared to normal rats, indicating that MIA induced impaired exercise ability and osteoarthritis development. However, oral administration of ibuprofen or salmon nasal cartilage-derived proteoglycans mitigated these impairments compared to MIA-injected rats that did not receive treatment (*p* < 0.05; Figure 1C,D). These findings indicate that oral administration of salmon nasal cartilage-derived proteoglycans potentially alleviates cartilage damage, pain, and impaired exercise ability associated with osteoarthritis.

### 2.2. Oral Administration of Salmon Nasal Cartilage-Derived Proteoglycans Suppresses MIA-Induced Inflammation in the Cartilage Tissue of Rats

**Figure 2:**
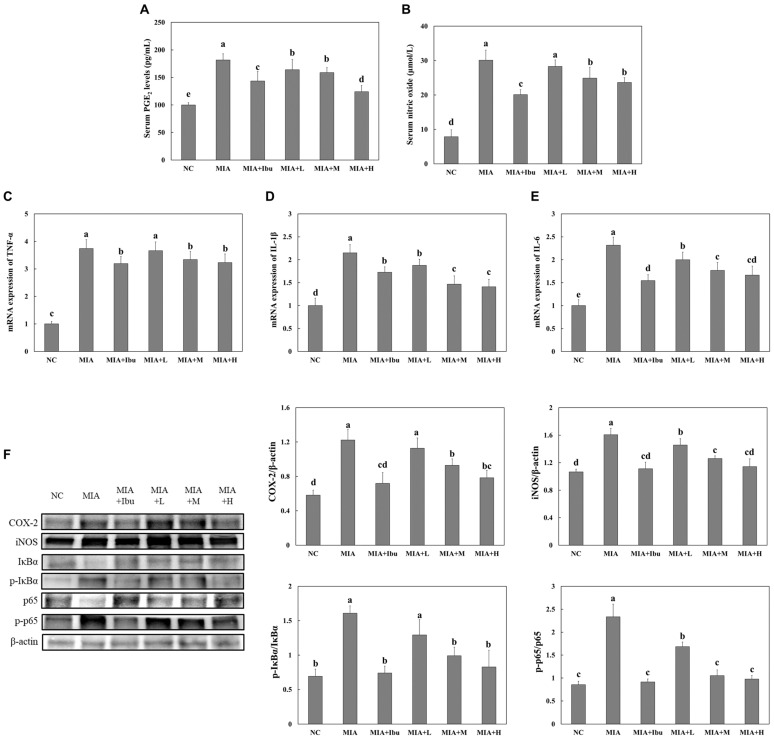
Effects of salmon nasal cartilage-derived proteoglycans on serum PGE_2_ (**A**), serum nitric oxide (**B**), mRNA expression of *TNF-α* (**C**), *IL-1β* (**D**), and *IL-6* (**E**), and protein expression of COX-2, iNOS, p-IκBα, p-p65 (**F**) in cartilage tissue from rats with MIA-induced osteoarthritis. Normal control (NC): normal diet; MIA: normal diet + MIA injection; MIA+Ibu: diet containing 20 mg/kg/bw ibuprofen + MIA injection; MIA+L: diet containing 2.1 mg/kg/bw salmon nasal cartilage-derived proteoglycans + MIA injection; MIA+M: diet containing 4.2 mg/kg/bw salmon nasal cartilage-derived proteoglycans + MIA injection; MIA+H: diet containing 8.4 mg/kg/bw salmon nasal cartilage-derived proteoglycans + MIA injection. Data represent the mean + SD. Different letters (a > b > c > d > e) indicate statistically significant differences between groups (*p* < 0.05), as determined using Duncan’s multiple range test.

MIA-injected rats exhibited significantly elevated serum levels of prostaglandin E_2_ (PGE_2_) and nitric oxide (NO) compared to normal rats, indicating the induction of inflammation. However, serum levels of PGE_2_ and NO were significantly lower in MIA-injected rats treated with ibuprofen or salmon nasal cartilage-derived proteoglycans compared to those that did not receive treatment (*p* < 0.05; Figure 2A,B). MIA injection also increased the mRNA expression of pro-inflammatory cytokines (Figure 2C–E) and the protein levels of COX-2, iNOS, phospho-IκBα/IκBα, and phospho-p65/p65 (Figure 2F) in the cartilage tissue. However, treatment with ibuprofen or salmon nasal cartilage-derived proteoglycans significantly reduced the levels of these inflammatory markers in the cartilage tissue of MIA-injected rats (*p* < 0.05). These results indicate that oral administration of salmon nasal cartilage-derived proteoglycans effectively suppresses osteoarthritis-related inflammation in the cartilage tissue.

### 2.3. Oral Administration of Salmon Nasal Cartilage-Derived Proteoglycans Suppresses MIA-Induced Cartilage Degradation Pathways in Rats

**Figure 3:**
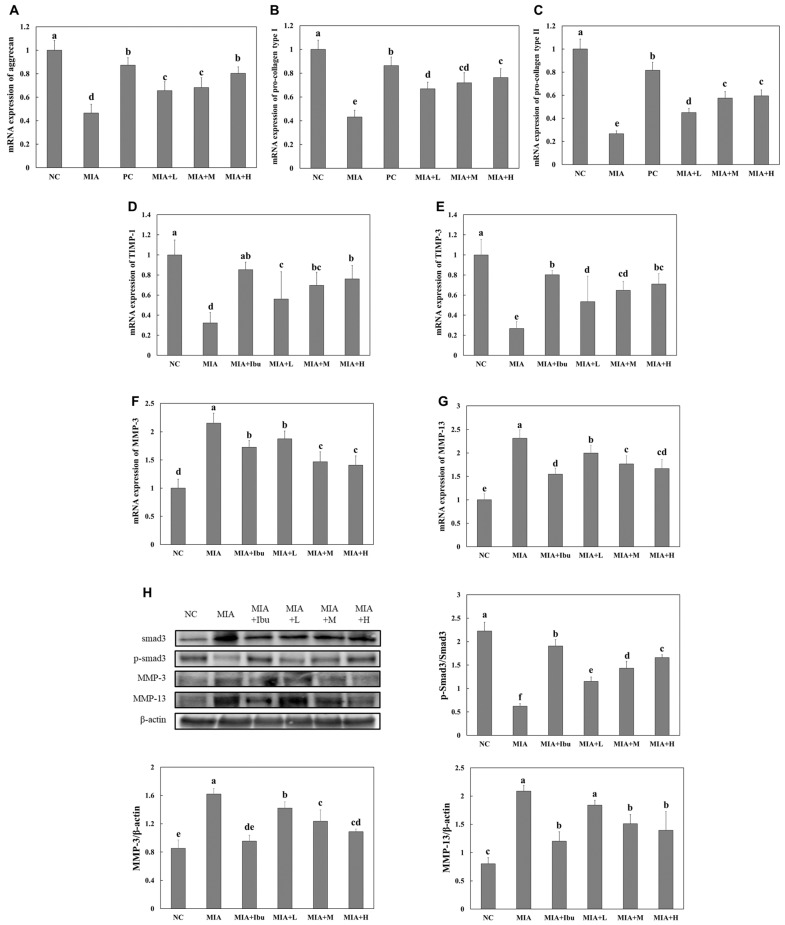
Effects of salmon proteoglycans on the mRNA expression of *aggrecan* (**A**), *pro-collagen type I* (**B**), *pro-collagen type II* (**C**), *TIMP-1* (**D**), *TIMP-3* (**E**), *MMP-3* (**F**), and *MMP-13* (**G**) and on the protein expression of p-Smad3 and MMPs (**H**) in cartilage tissue from rats with MIA-induced osteoarthritis. Normal control (NC): normal diet; MIA: normal diet + MIA injection; MIA+Ibu: diet containing 20 mg/kg/bw ibuprofen + MIA injection; MIA+L: diet containing 2.1 mg/kg/bw salmon proteoglycans + MIA injection; MIA+M: diet containing 4.2 mg/kg/bw salmon proteoglycans + MIA injection; MIA+H: diet containing 8.4 mg/kg/bw salmon proteoglycans + MIA injection. Data represent the mean + SD. Different letters (a > b > c > d > e > f) indicate statistically significant differences between groups (*p* < 0.05), as determined using Duncan’s multiple range test.

The mRNA expression levels of extracellular matrix components, such as aggrecan, collagen type I, and collagen type II, were significantly reduced in the cartilage tissue of MIA-injected rats compared to normal rats. Additionally, the mRNA levels of tissue inhibitors of metalloproteinases (TIMP)-1 and TIMP-3 were notably lower, while those of MMP-3 and MMP-9 were significantly higher in MIA-injected rats compared to normal rats. However, MIA-injected rats treated with ibuprofen or salmon nasal cartilage-derived proteoglycans exhibited significantly increased mRNA levels of aggrecan, collagen type I, collagen type II, TIMP-1, and TIMP-3 and decreased mRNA levels of MMP-3 and MMP-9 compared to MIA-injected rats that did not receive treatment (*p* < 0.05; Figure 3A–G). Furthermore, MIA-injected rats treated with ibuprofen or salmon nasal cartilage-derived proteoglycans exhibited significantly increased levels of Smad3 phosphorylation proteins and decreased levels of MMP-3 and MMP-9 proteins in the cartilage tissue compared to MIA-injected rats that did not receive treatment. These results suggest that salmon nasal cartilage-derived proteoglycans may help mitigate osteoarthritis by activating Smad3 and suppressing MMPs, thereby influencing the synthesis and degradation of extracellular matrix components in articular cartilage.

### 2.4. Oral Administration of Salmon Nasal Cartilage-Derived Proteoglycans Suppresses MIA-Induced Apoptosis in the Articular Cartilage of Rats

**Figure 4:**
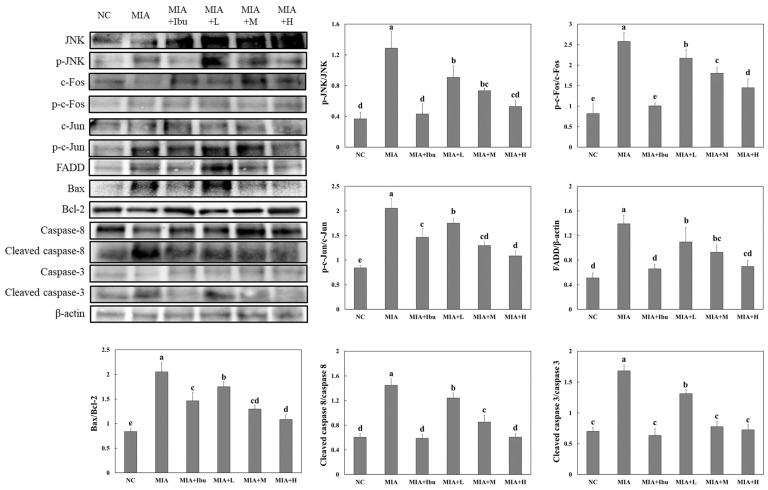
Effects of salmon proteoglycans on the protein expression of p-JNK, p-c-Fos, p-Jun, FADD, Bax, Bcl-2, cleaved caspase 8, and cleaved caspase 3 in cartilage tissue from rats with MIA-induced osteoarthritis. Normal control (NC): normal diet; MIA: normal diet + MIA injection; MIA+Ibu: diet containing 20 mg/kg/bw ibuprofen + MIA injection; MIA+L: diet containing 2.1 mg/kg/bw salmon proteoglycans + MIA injection; MIA+M: diet containing 4.2 mg/kg/bw salmon proteoglycans + MIA injection; MIA+H: diet containing 8.4 mg/kg/bw salmon proteoglycans + MIA injection. Data represent the mean + SD. Different letters (a > b > c > d > e) indicate statistically significant differences between groups (*p* < 0.05), as determined using Duncan’s multiple range test.

The loss of chondrocytes through apoptosis diminishes the cell’s ability to synthesize and repair the extracellular matrix, accelerating cartilage degeneration. A series of experiments were conducted to evaluate the effects of salmon nasal cartilage-derived proteoglycans on apoptotic factors in articular cartilage. As shown in Figure 4, MIA injection activated apoptosis signaling pathways, including the JNK/c-Fos and c-Jun pathways, as well as the FADD/caspase8/Bax/caspase3 pathway in the articular cartilage of MIA-injected rats. Notably, MIA-injected rats treated with ibuprofen or salmon nasal cartilage-derived proteoglycans exhibited significantly reduced expression levels of proteins involved in these apoptosis pathways compared to MIA-injected rats that did not receive treatment (*p* < 0.05). These findings indicate that oral administration of salmon nasal cartilage-derived proteoglycans can protect articular cartilage by inhibiting chondrocyte apoptosis.

## 3. Discussion

The extraction of proteoglycans from salmon nasal cartilage is a well-established process, leveraging the abundant availability of salmon by-products from the fishing industry. Advances in extraction technology have enabled the efficient and cost-effective production of high-purity proteoglycans [15,16]. Currently, the utilization and industrial application of proteoglycans are advancing rapidly, with a growing body of evidence supporting their efficacy and safety. The development of proteoglycan-based products spans various categories, including dietary supplements, functional foods, and medical devices. Continued research and development, coupled with strategic commercialization efforts, will be crucial for realizing the full potential of proteoglycans in joint health and beyond [17]. The present study aimed to evaluate the efficacy of salmon nasal cartilage-derived proteoglycans in mitigating osteoarthritis symptoms using an experimental animal model. The results provide compelling evidence supporting the therapeutic potential of salmon nasal cartilage-derived proteoglycans in osteoarthritis management. Specifically, the oral administration of salmon nasal cartilage-derived proteoglycans improved cartilage integrity, reduced inflammation, suppressed cartilage degradation pathways, and inhibited chondrocyte apoptosis.

The results showed that MIA injection significantly increased the serum levels of PGE_2_ and NO, as well as the expression of pro-inflammatory cytokines and proteins involved in inflammatory responses within the cartilage tissue. These compounds play pivotal roles in the inflammatory processes associated with osteoarthritis. This aligns with the existing literature highlighting the role of inflammatory mediators in the pathogenesis of osteoarthritis [18,19,20]. Notably, salmon nasal cartilage-derived proteoglycans significantly reduced these inflammatory markers, similar to the effects observed with ibuprofen, a known anti-inflammatory agent, in rats with MIA-induced osteoarthritis. These findings suggest that salmon nasal cartilage-derived proteoglycans possess potent anti-inflammatory properties, which are crucial for alleviating osteoarthritis symptoms by reducing swelling and pain in the affected joints.

Proteoglycans contribute to maintaining the structural integrity of cartilage by modulating the balance between the synthesis and degradation of extracellular matrix components [12]. In the present study, MIA injection disrupted this balance, resulting in decreased expression of essential extracellular matrix components, including aggrecan, collagen type I, and collagen type II, while simultaneously increasing the levels of MMPs like MMP-3 and MMP-9. These changes contributed to the breakdown of the extracellular matrix and subsequent cartilage deterioration, consistent with the existing literature [21,22,23,24]. Notably, the administration of salmon nasal cartilage-derived proteoglycans reversed these effects, increasing extracellular matrix component synthesis and TIMP expression while reducing MMP levels. These observations indicate that salmon nasal cartilage-derived proteoglycans can effectively mitigate cartilage degradation by restoring the balance between the synthesis and degradation of extracellular matrix components.

Chondrocytes are essential for the maintenance and repair of cartilage. Chondrocyte apoptosis plays a pivotal role in the progression of osteoarthritis by diminishing the cell’s capacity for extracellular matrix synthesis and repair [11]. The results showed that MIA injection activated several apoptosis signaling pathways in chondrocytes, consistent with the known mechanisms by which inflammatory and stress-related signals promote chondrocyte apoptosis in osteoarthritis. Furthermore, treatment with salmon nasal cartilage-derived proteoglycans significantly reduced the expression of apoptosis-related proteins in these pathways, suggesting a protective effect against chondrocyte apoptosis. This anti-apoptotic action likely contributes to the preservation of cartilage tissue and overall joint function.

Throughout this study, the effects of salmon nasal cartilage-derived proteoglycans were compared with those of ibuprofen, a widely used nonsteroidal anti-inflammatory drug (NSAID) for osteoarthritis management [25]. Notably, both treatments showed similar efficacy in reducing inflammation, cartilage degradation, and chondrocyte apoptosis, indicating that salmon nasal cartilage-derived proteoglycans may serve as an effective alternative or adjunct to traditional NSAIDs in osteoarthritis therapy. Additionally, the potentially lower side effect profile of salmon nasal cartilage-derived proteoglycans compared to NSAIDs offers a significant advantage for the long-term management of osteoarthritis.

The findings of this study underscore the significant potential of salmon nasal cartilage-derived proteoglycans in managing osteoarthritis. By reducing inflammation, preventing cartilage degradation, and inhibiting chondrocyte apoptosis, these bioactive compounds offer a promising therapeutic strategy for osteoarthritis. However, this study is limited by its reliance on an animal model, which may not fully represent human osteoarthritis, and the specific dosage used may not directly translate to optimal dosages for human treatment. Additionally, the long-term safety and efficacy of salmon nasal cartilage-derived proteoglycans remain to be established. Future studies should focus on the detailed molecular pathways influenced by salmon nasal cartilage-derived proteoglycans to better understand their protective mechanisms against osteoarthritis. Given their anti-inflammatory and chondroprotective properties, salmon nasal cartilage-derived proteoglycans may also hold potential for managing other inflammatory joint disorders.

## 4. Materials and Methods

### 4.1. Preparation of Salmon Nasal Cartilage-Derived Proteoglycans

**Figure 5:**
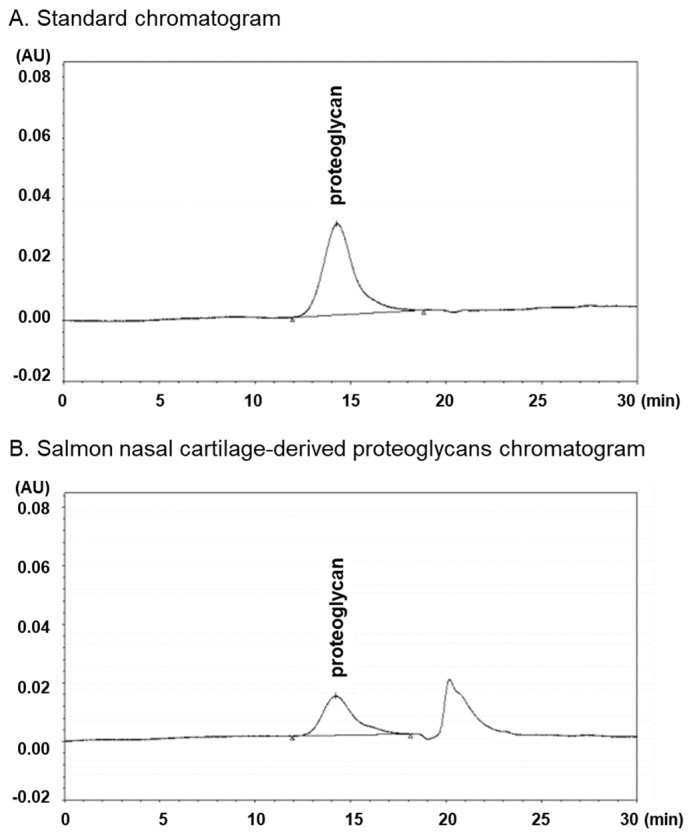
High-performance liquid chromatography analysis of proteoglycans in salmon nasal cartilage-derived proteoglycans. Standard (**A**) and salmon nasal cartilage-derived proteoglycans (**B**).

Salmon nasal cartilage-derived proteoglycans (Proteoglycan HG-100) were supplied from Nihon Pharmaceutical Co., Ltd. (Tokyo, Japan). The salmon nasal cartilage-derived proteoglycans were found to contain 41.935 ± 0.92% of proteoglycans, as measured by high-performance liquid chromatography (HPLC) (Figure 5).

### 4.2. Animal Model and Treatment

Male Sprague–Dawley rats (6–8 weeks old) were used in this study. Osteoarthritis was induced by a single intra-articular injection of MIA (50 μL, 60 mg/mL) into the knee joint. Control rats received an equivalent volume of saline. The animals were randomly assigned to one of six groups: normal control (NC), MIA-induced osteoarthritis control (MIA), MIA + ibuprofen (20 mg/kg/day, MIA+Ibu), and MIA + salmon nasal cartilage-derived proteoglycans (2.1 mg/kg/day, MIA-L; 4.2 mg/kg/day, MIA-M; 8.4 mg/kg/day, MIA-H). Treatments were administered orally via gavage once daily for 31 days.

The Institutional Animal Care and Use Committee of Kyung Hee University approved the protocol (KHGASP-24-433) for the animal studies. The animals were maintained in accordance with the university’s “Guidelines for Animal Experiments”.

### 4.3. Assessment of Exercise Ability

Exercise ability was evaluated using a treadmill test (Jeollanamdo Institute of Natural Resources Research, Jeonnam Naju City, Republic of Korea). Parameters measured included rear pressure, rear propel, and running speed. Each rat was acclimated to the treadmill before testing, and their exercise performance was recorded.

### 4.4. Histological Observation

At the end of the treatment period, rats were euthanized, and knee joints were harvested for histological analysis. The joints were fixed in 10% formalin, decalcified, and embedded in paraffin. Sections (5 µm) were stained with hematoxylin and eosin (H&E) for morphological evaluation and assessment of cartilage degradation.

### 4.5. Micro-Computed Tomography

Bone mineral density, bone volume/total tissue volume, trabecular number, trabecular thickness, and trabecular separation were assessed using a micro-CT scanner (Skyscan 1176, Bruker, Belgium). Scans of the knee joints were performed, and the resulting images were analyzed using software.

### 4.6. Serum Assays

At the end of the treatment period, blood samples were collected from the rats. Serum levels of PGE_2_ and NO were measured using ELISA kits (R&D Systems, Minneapolis, MN, USA), following the manufacturer’s instructions.

### 4.7. Total RNA Isolation and Real-Time PCR

Cartilage tissue isolated from the knee joints was used for mRNA extraction. mRNA expression levels of the *TNF-α*, *IL-1β*, *IL-6*, *aggrecan*, *pro-collagen type I*, *collagen type I*, *TIMP-1*, *TIMP-3*, *MMP-3*, *MMP-13*, and *GAPDH* genes were analyzed using real-time PCR, following previously described methods [26].

### 4.8. Protein Extraction and Western Blot Analysis

Cartilage tissue isolated from the knee joints was used for protein extraction. Western blot analysis was conducted to assess the expression levels of COX-2, iNOS, IkB-α, phospho-IkB-α, p65, phospho-p65, smad3, MMP-3, MMP-13, JNK, phospho-JNK, c-FOS, phospho-c-FOS, c-Jun, phospho-c-Jun, FADD, Bax, Bcl-2, caspase-8, caspase-3, and β-actin proteins, following previously described methods [26].

### 4.9. Statistical Analysis

Data are expressed as the mean ± SD. Statistical analysis was performed using one-way ANOVA, followed by Duncan’s multiple-range test for post-hoc comparisons. A *p*-value of <0.05 was considered statistically significant.

## 5. Conclusions

The present study demonstrates that oral administration of salmon nasal cartilage-derived proteoglycans holds significant therapeutic potential for mitigating osteoarthritis symptoms. Salmon nasal cartilage-derived proteoglycans effectively improved cartilage morphology, enhanced bone mineral density, and restored exercise ability in rats with MIA-induced osteoarthritis. These findings suggest that salmon nasal cartilage-derived proteoglycans can protect articular cartilage by modulating inflammatory responses, preventing matrix degradation, and promoting cell survival, with efficacy comparable to ibuprofen. Overall, this study highlights the potential of salmon nasal cartilage-derived proteoglycans as a viable alternative or adjunct therapy for managing osteoarthritis. Future studies should focus on long-term efficacy, optimal dosing regimens, and potential synergistic effects with other therapeutic agents.

## Figures and Tables

**Table 1:** Effects of salmon nasal cartilage-derived proteoglycans on the bone marrow density, bone volume/total tissue volume, trabecular number, trabecular thickness, and trabecular separation of rats with MIA-induced osteoarthritis, as determined using micro-CT analysis.

Measurements	NC	MIA	MIA+Ibu	MIA+L	MIA+M	MIA+H
Bone marrow density (mg/cc)	323.29 ± 16.30 ^a^	236.10 ± 29.57 ^e^	304.15 ± 11.11 ^ab^	259.57 ± 32.67 ^de^	275.75 ± 7.75 ^cd^	286.63 ± 8.14 ^bc^
Bone volume/ Total tissue volume	0.38 ± 0.02 ^a^	0.15 ± 0.03 ^d^	0.33 ± 0.06 ^b^	0.19 ± 0.04 ^d^	0.24 ± 0.02 ^c^	0.27 ± 0.01 ^c^
Trabecular number	1.15 ± 0.09 ^a^	1.04 ± 0.06 ^e^	1.41 ± 0.07 ^b^	1.08 ± 0.04 ^d^	1.13 ± 0.07 ^d^	1.24 ± 0.09 ^c^
Trabecular thickness (mm)	0.28 ± 0.02 ^a^	0.13 ± 0.0 ^e^	0.25 ± 0.03 ^b^	0.19 ± 0.03 ^d^	0.21 ± 0.01 ^cd^	0.22 ± 0.01 ^c^
Trabecular separation (mm)	0.46 ± 0.03 ^d^	0.64 ± 0.04 ^a^	0.49 ± 0.03 ^cd^	0.57 ± 0.05 ^b^	0.57 ± 0.04 ^b^	0.53 ± 0.03 ^bc^

Normal control (NC): normal diet; MIA: normal diet + MIA injection; MIA+Ibu: diet containing 20 mg/kg/bw ibuprofen + MIA injection; MIA+L: diet containing 2.1 mg/kg/bw salmon nasal cartilage-derived proteoglycans + MIA injection; MIA+M: diet containing 4.2 mg/kg/bw salmon nasal cartilage-derived proteoglycans + MIA injection; MIA+H: diet containing 8.4 mg/kg/bw salmon nasal cartilage-derived proteoglycans + MIA injection. Data represent the mean + SD. Different letters (a > b > c > d > e) indicate statistically significant differences between groups (*p* < 0.05), as determined using Duncan’s multiple range test.

## Data Availability

The data presented in this study are available on request from the corresponding author.
